# Starch phosphorylation—A needle in a haystack

**DOI:** 10.1186/s13007-024-01237-9

**Published:** 2024-07-27

**Authors:** Julia Compart, Ardha Apriyanto, Joerg Fettke

**Affiliations:** 1 Biopolymer Analytics, Institute of Biochemistry and Biology, University of Potsdam, Karl- Liebknecht-Str. 24-25, Building 20, Potsdam-Golm, Germany; 2 Research and Development, PT. Astra Agro Lestari Tbk. Jl. Puloayang Raya Blok OR I, Kawasan Industri Pulogadung, Jakarta Timur, Indonesia

**Keywords:** Starch phosphorylation, Starch, Phosphoglucans, Glucan, α-glucan, water dikinase, Mass spectrometry, MALDI-fTOF MS, MS/MS, Reducing ends

## Abstract

Phosphoesterification is the only naturally occurring covalent starch modification identified to date, and it has a major impact on overall starch metabolism. The incorporation of phosphate groups mediated by dikinases [α-glucan, water dikinase (GWD), EC 2.7.9.4; phosphoglucan, water dikinase (PWD), EC 2.7.9.5] massively alters the starch granule properties; however, previous studies did not determine whether the starch-related dikinases bind the phosphate to the glucosyl units within the amylopectin molecules in a specific pattern or randomly. In order to answer this challenging question, a number of approaches were initially pursued until a protocol could be established that enabled a massive step forward in the in vitro analysis of phosphorylated glucan chains obtained from starch. For this purpose, phosphorylation by GWD was investigated, including the final state of phosphorylation i.e., the state of substrate saturation when GWD lacks further free hydroxyl groups at OH-C6 for the catalysis of monophosphate esters. Since the separated phosphorylated glucan chains were required for the analysis, isoamylase digestion was performed to cleave the α-1,6-glycosidic bonds and to allow for the removal of the huge number of existing neutral chains by means of anion exchange chromatography. Via Matrix-Assisted Laser Desorption/Ionization–Time of Flight (MALDI-TOF) MS and MALDI-MS/MS, the phosphorylated α-glucan chains were analysed, and the position of the phosphate group within the chain in relation to the reducing end was determined. Here, we demonstrate a protocol that enables the analysis of phosphorylated oligosaccharides, even in small quantities.

## Background

Starch covers a large part of our daily calorie requirements, which is of particular interest in various food applications, however, due to its special structural properties, starch is being used in many areas of the non-food industry as well. In plants, starch is the end product of photosynthesis and serves as a chemical storage form of solar energy. It consists of two types of polysaccharides, amylose and amylopectin, composed of linear α-1,4-linked and branched α-1,6-linked D-glucosyl residues. However, compared to amylose, amylopectin has a considerably higher branching frequency of around 5% [1]. These branching points within amylopectins are clustered, causing adjacent chains of amylopectin molecules to form double helices, which are organized in water-insoluble semi-crystalline granules [2, 3]. The phosphoesterification is the only known natural covalent starch modification, with an enormous impact on both starch degradation and synthesis [4, 5]. It has been suggested that the amylopectin is exclusively phosphorylated by two starch-related dikinases: the α-glucan, water dikinase [GWD, EC 2.7.9.4] [6] and the phosphoglucan, water dikinase [PWD, EC 2.7.9.5] [7, 8]. These attach the phosphate groups to OH-C6 and OH-C3, respectively. The introduction of the phosphate groups results in charges that impair the hydrogen bonding of the amylopectin double helices, leading to an opening of the double helical structures and a reduction in crystallinity, i.e., a phase transition of the glucans from a highly ordered to a solubilized state [9]. These structural alterations enable the action of downstream-acting proteins (e.g., β-amylase [BAM, EC 3.2.1.2] [10], isoamylase [ISA, EC 3.2.1.68] [11], starch synthases [SS, EC 2.4.1.21] [12], and α-1.4-glucan phosphorylase 1 [PHS1, EC 2.4.1.1] [13]) of the starch turnover pathways. It has been shown that the phosphate content of starch from different tissues, organs, developmental stages, and species is highly variable [2]. It has also been reported that the presence of phosphates in the starch granules can influence parameters as the degree of crystallinity, the inner structure and the granule diameter, e.g.for potato tubers, a negative correlation between the phosphate content and granule diameter was observed [14, 15]. An opposite effect was found in the *Arabidopsis thaliana sex4/dpe2* mutant, in which larger granules showed a higher phosphate content than smaller ones [5]. However, GWD-mediated phosphorylation was shown to have a pronounced effect on starch degradation, morphology, and structure [16]. Transgenic *GWD* overexpression lines of different crops showed improvements in multiple key traits, such as yield, grain shape, quality, seed germination, and stress tolerance [17, 18]. The phosphate content has a pronounced impact on the physico-chemical properties of starch, such as its hydrophilicity, surface charge, chemical susceptibility, and crystallinity; consequently, it also influences properties such as the thermal stability, clarity, viscosity, digestibility, and swelling power. The altered starch properties, in turn, have an effect on the possible areas of application. Industry is increasingly interested in minimizing the processing of raw materials in order to save costs. It is therefore of great interest to further characterize the molecular mechanism of starch phosphorylation [19, 20].

Our research group has conducted several studies to analyse phosphorylation in depth. We previously demonstrated that the action of GWD leads to the formation of single and double phosphorylated glucan chains when using native starch granules [21] and even triple phosphorylated chains when using highly crystalline maltodextrin [9, 22]. However, the position within the glucan chain where the phosphate group was covalently attached, and whether this occurred according to a specific or a random distribution pattern, remained obscure. Furthermore, it is unknown as to whether this phosphorylation distribution is conserved for starches from various species, mutants, and tissues and *vice versa* for starch-related dikinases of the plant kingdom. Answering these questions is quite challenging, as only a small proportion of the total glucan chains of starch granules are phosphorylated, and the immense abundance of neutral chains interferes with analyses. Here, we demonstrate an established protocol to analyse single phosphorylated glucan chains of starches in detail using recombinant StGWD (from *Solanum tuberosum* L.). We combined various in vitro assays with different enzymes and mass spectrometric analyses that allowed us to determine the position of the phosphate groups in relation to the reducing ends. In addition, we were able to track phosphate incorporation using radioactive labelling, which ensured the validation of the individual protocol steps due to its high sensitivity.

## Methods

### Reagents

All chemicals were purchased from Sigma-Aldrich (München, Germany) or Carl Roth (Karlsruhe, Germany). Isoamylase from Pseudomonas *sp.* and β-amylase from barley were obtained from Megazyme (Bray, Ireland). The [β-^33^P]ATP (3000 Ci mmol^− 1^) used in the experiments was purchased from Hartmann Analytic (Braunschweig, Germany). Maize *amylose extender(ae)* starch was acquired from ICN Biomedicals (Eschborn, Germany).

The expression and purification of recombinant GWD from potato (*Solanum tuberosum* L.) were performed according to the procedure described by Hejazi, Steup, and Fettke, 2012 [23].

### Reagent setup

**Reaction buffer for GWD-mediated in vitro** **starch phosphorylation**.

50 mM Hepes, pH 7.5 (adjusted with NaOH);

1 µCi [β-³³P]-ATP;

2 mM EDTA;

6 mM MgCl₂;

2 mM DTE;

0.4 mg*ml^− 1^ BSA;

25 µM ATP.

### Isoamylase digestion

10 mM ammonium acetate, pH 4.5 (adjusted with acetic acid).

β-amylase digestion.

16 mM ammonium acetate, pH 6.0 (adjusted with acetic acid).

### Matrix preparation for MALDI-TOF MS analysis

First, prepare a stock solution of p-coumaric acid (CA):

5 mg CA in 300 µl acetonitrile/water (50:50, v/v) and 2 mM ammonium dihydrogen phosphate.

Next, prepare the 3-aminoquinoline/p-coumaric acid (AQ/CA) matrix:

10 mg AQ in 75 µl CA.

### Procedure

#### in vitro starch phosphorylation

**Fig. 1:**
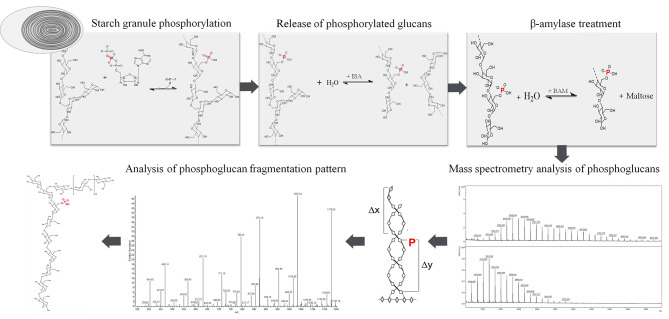
Schematic representation of the procedure for analysing phosphorylated starches/glucans. Starch granules are phosphorylated in vitro with a starch-related dikinase. Subsequently, the starch is boiled and hydrolysed with isoamylase. In addition, a β-amylase digestion step is performed to determine the position of the phosphate group within the glucan chain. The phosphorylated chains are analysed using MALDI-TOF MS and MALDI-MS/MS

First, the maize starch granules were prepared for in vitro phosphorylation by GWD. To this end, 400 mg of starch was added to reaction tubes, then washed thoroughly with water and once with reaction buffer containing 50 mM Hepes/NaOH (pH 7.5), 2 mM EDTA, 6 mM MgCl₂, 2 mM DTE, 0.4 mg*ml^− 1^ BSA, and 25 µM ATP to set the appropriate conditions. Next, fresh reaction buffer with 1 µCi [β-^33^P]ATP was applied to the starch and mixed by means of vigorous shaking; then, 2 µg GWD was added. The mixtures were incubated for 1 h at 30 °C with vigorous shaking. Afterwards, the samples were centrifuged at 8000 g for 1 min at room temperature, and the supernatant was discarded. Than reaction buffer with 1 µCi [β-^33^P]ATP and GWD was added again and the incubation was repeated as described above. After the second incubation step, the phosphorylated starch granules were centrifuged again, and the supernatant was discarded. The starch was washed in a 50 mL Falcon tube with 40 ml of water six to ten times until the unbound ³³P was eliminated. To prove that the unbound ATP was sufficiently washed away, 1 mL of the wash supernatant was used for liquid scintillation counting each time. Following the washing step, 10 mL of water was added to the starch pellet, and an aliquot of 200 µL was applied to 3 mL of the scintillation cocktail to measure the ³³P incorporation (Fig. 1).

### Release of phosphoglucans by isoamylase digestion

For ISA digestion, the GWD-treated starch granules were boiled in a water bath at 100 °C for 10 min with constant agitation until the starch was partly solubilized. After boiling, we waited until the sample had cooled down to 50 °C, and then the ammonium acetate buffer (10 mM final concentration, pH 4.5) and 4 U ISA were applied. The sample was incubated overnight at 50 °C under continuous agitation. The following day, the sample was centrifuged at 10,000 g for 10 min at room temperature, and the supernatant containing the phosphoglucans was separated from the pellet. Aliquots of the supernatant and the pellet were utilized for scintillation counting (Fig. 1).

### Purification and enrichment of the phosphorylated glucan chains

A column (column volume 2.3 mL, reservoir volume 3.2 mL, column length 10 cm, column diameter 2.15 cm) containing 2 mL Q Sepharose FF as the matrix was connected to a peristaltic pump, and a flow rate of 1 mL/min was set. A prewash of the column with 10 cv of water and equilibration with 15 cv of 1 M ammonium acetate were performed. Then, the column was washed again with 10 cv of water. The supernatant from the isoamylase hydrolysis step was applied to the column, and the column was washed with 30 cv of water. After thorough washing, the phosphorylated glucans were enriched in a four-step elution with increasing ammonium acetate concentrations at pH 6.5 (2 cv 0.01 M ammonium acetate, 2 cv 0.02 M ammonium acetate, 4 cv 0.2 M ammonium acetate, and 2 cv 1 M ammonium acetate). One-millilitre fractions were collected. From each fraction, 20 µL was taken and mixed with 3 mL of scintillation cocktail to detect the ^33^P content by means of scintillation counting. According to the scintillation counting results, another aliquot corresponding to approximately 300 cpm was kept for thin-layer chromatography (TLC).

### Detection of phosphorylated glucans *via* thin-layer chromatography

The aliquots were dried and washed three times with 50 µL water each time using a vacuum concentrator. Afterwards, 1.5 µL of 250 mM ammonium bicarbonate was added to each fraction.

Ammonium bicarbonate (250 mM) was added to the developing chamber to form the mobile phase; we then waited for around 30 min, until the gas phase was completely saturated with the solvent.

From each fraction, 1.5 µL was spotted onto a PEI cellulose plate; as a control, 0.05 µCi [β-^33^P]ATP was used. After the samples had dried, the plate was transferred to the developing chamber. The plate was removed from the developing chamber when the mobile phase was close to the top. The dried thin-layer plate was placed together with a phosphor imaging plate (FUJI MS 40,120,111) overnight in a cassette. The next day, the ³³P compounds were recorded by means of phosphor imaging (FLA-3000, Fujifilm) (Fig. 1).

### β-amylolysis of phosphorylated glucans

According to the TLC results, all fractions containing phosphoglucans were divided evenly into two aliquots and dried in a vacuum centrifuge. One aliquot was digested with β-amylase, and the other was kept exclusively treated with isoamylase. For the β-amylase digestion, 100 µL of ammonium acetate buffer (16 mM final concentration, pH 6) and 1 U of β-amylase were added to one of the aliquots of each fraction and incubated for 3 h at 50 °C. Afterwards, another 100 µL of ammonium acetate buffer and 1 U of β-amylase were added, and the samples were incubated overnight. The next day, the samples were dried in the vacuum centrifuge (Fig. 1).

### Labelling of phosphoglucans

The purified phosphoglucans were resuspended in 20 µl of water. From each fraction and aliquot, 0.5 µL was spotted onto the MALDI-MS target, together with 0.5 µL of the 3-aminoquinoline/p-coumaric acid matrix (3AQ/CA; 1.67% [w/v] CA in 50% ACN [v/v] and 2 mM ammonium dihydrogen phosphate, 13.3% [w/v] 3AQ in CA solution). The target was kept at 60 °C for 1 h in order to label the reducing ends of the phosphoglucans with 3AQ (Fig.1).

### Detection of phosphoglucans

For MS analyses, a Microflex LRF and an Autoflex (Bruker Daltonik, Bremen, Germany) were used in the positive and negative ion reflector modes. For MS/MS, a Thermo LTQ XL (Thermo Fisher Scientific, Waltham, USA) was utilized in the positive and negative ion modes (Fig.1).

## Results and discussion

### Enrichment of phosphorylated α-glucan chains

**Fig. 2:**
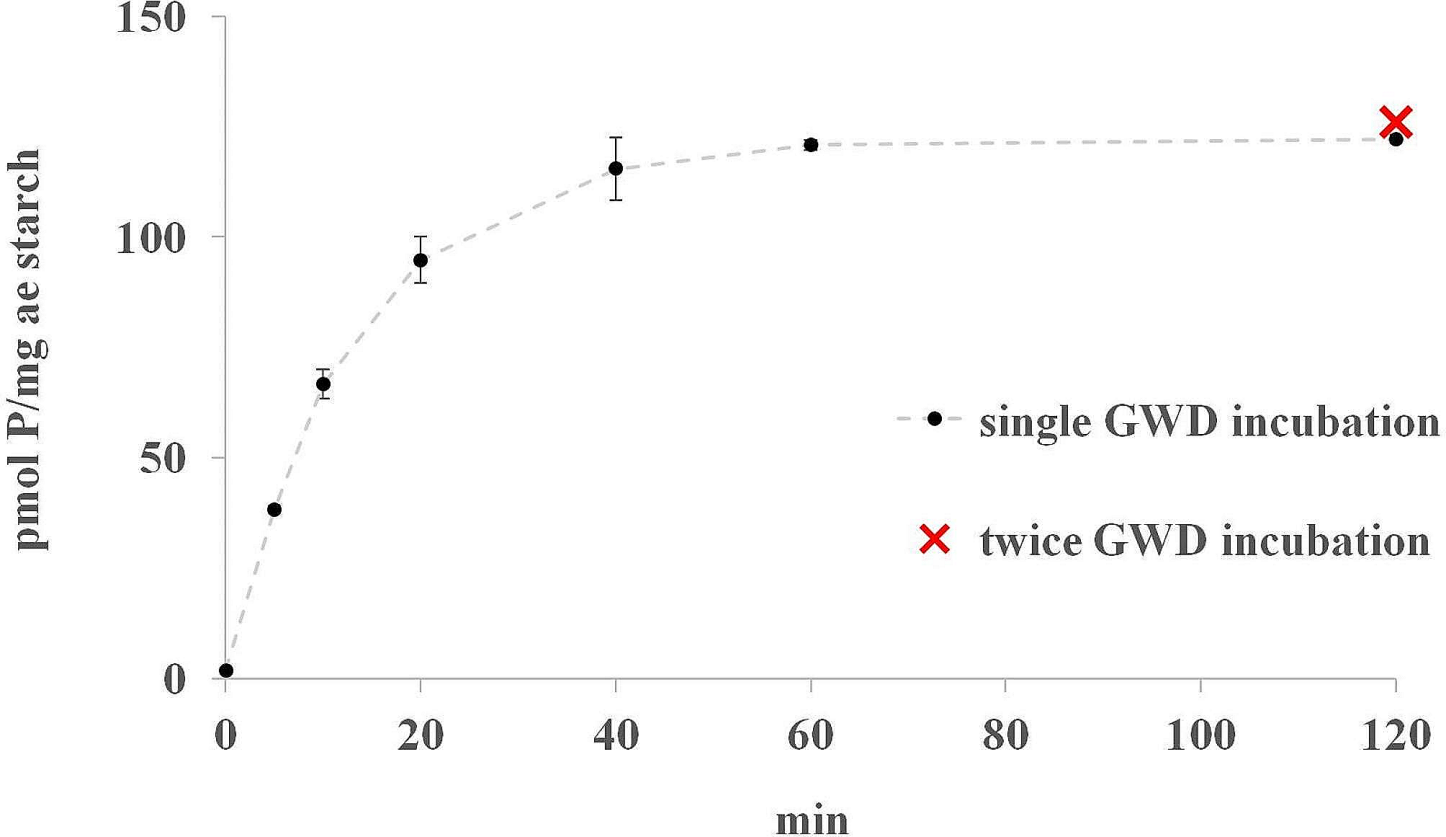
Phosphorylation of maize *ae* starch by StGWD. Maize *ae* starch was incubated with GWD and the ^33^P incorporation was determined at different time points within a two-hour time frame. Therefore, aliquots of 10 mg were taken after 0, 5, 10, 20, 40, 60 and 120 min and the reactions were stopped by adding 2% (w/v) SDS (black dashed line). Values are the means ± SD (*n* = 3). The starch was treated with GWD twice for one hour each (red x). GWD-mediated phosphate incorporation was monitored by radioactive labelled of [β-^33^P]ATP

In order to investigate the phosphorylation pattern, it was initially required to phosphorylate the starch in vitro with GWD. Therefore, maize *amylose extender* starch was chosen as it displayed a high level of phosphate incorporation when treated with *Solanum tuberosum L*. GWD [9, 24]. Furthermore, a previous study of phosphate distribution in different starches suggests an inverse relationship between branching frequency of amylopectin and total phosphate content, with phosphate tending to preferentially accumulate in longer, less branched α-glucan chains [25]. The commercial starch granules were washed sufficiently with water to remove the soluble sugars, which would inhibit the action of GWD. The incubation was repeated twice for one hour each time followed by starch heat-solubilisation and ISA hydrolysis. Here, it was crucial to boil the starch in a sufficient amount of water under constant shaking to prevent gelatinization, which would hinder the isoamylase and the release of phosphorylated glucans. After incubating the starch with GWD for around an hour, phosphorylation saturation (about 125 pmol phosphate per mg starch) was already achieved., i.e., the highest possible degree of phosphorylation of the starch granules (Fig. 2). However, depending on the starch origin, the overall phosphate incorporation and the incubation time can vary [26].

**Fig. 3:**
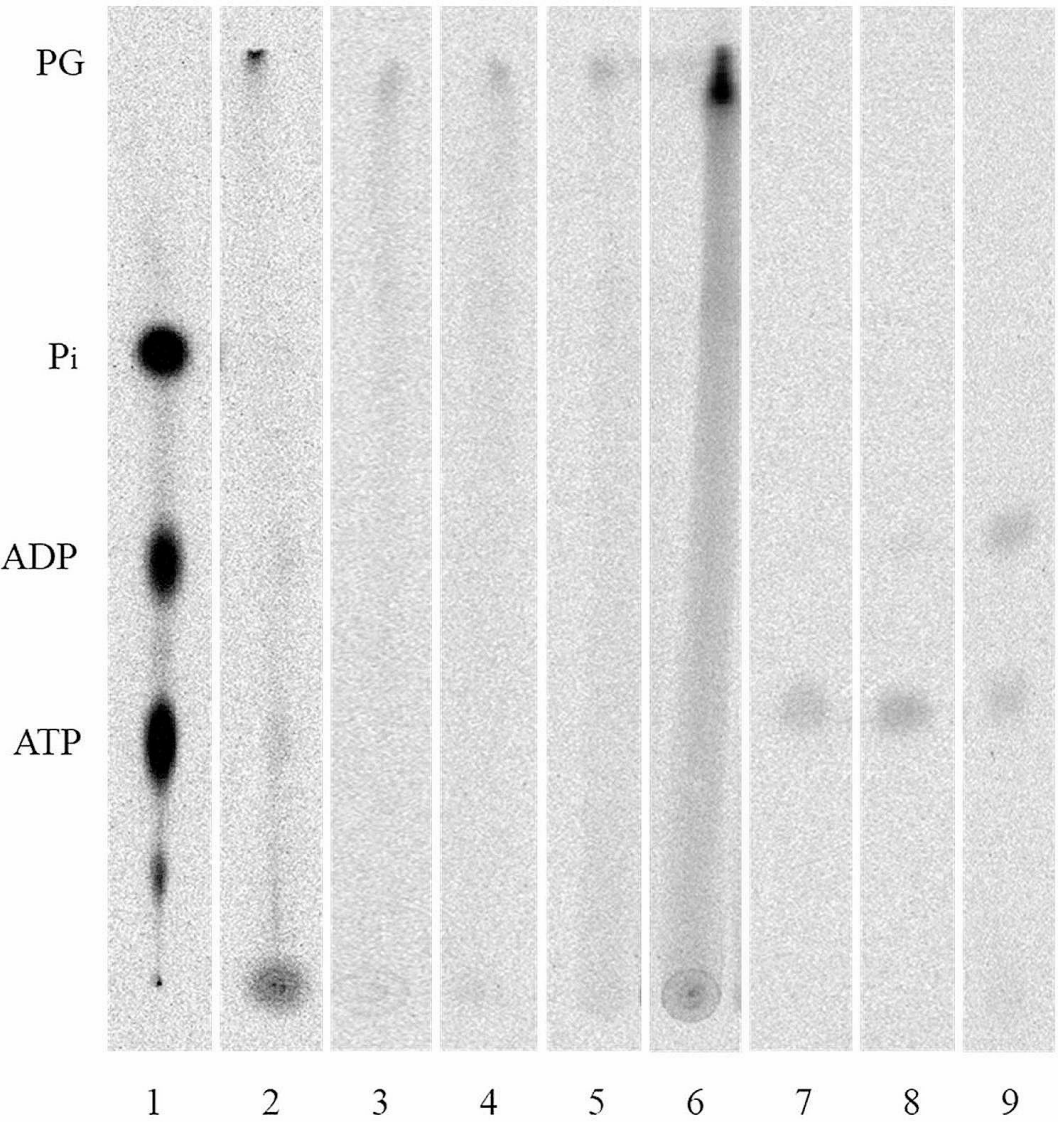
Thin-layer chromatography analysis of phosphorylated starch granules after isoamylase treatment (2) and anion exchange chromatography (3–9) Maize *ae* starch was incubated with StGWD and [β-^33^P]ATP. Afterwards, the starch was washed, boiled and treated by ISA. (1) mixture of [β-^33^P]ATP, [β-^33^P]ADP and ^33^P_i_ and (2) GWD- and ISA-treated starch granules were spotted onto a PEI cellulose plate, and the ^33^P-labelled components were analysed by means of phosphor imaging. (3–9) After anion exchange chromatography an aliquot of each fraction with a radioactive signal detected *via* scintillation counting was evaporated and analysed by means of TLC. The ^33^P signals were monitored by means of phosphor imaging. The results are displayed for (3–4) fractions eluted with 0.02 M ammonium acetate, (5–6) fractions eluted using 0.2 M ammonium acetate, and (7–9) fractions eluted using 1 M ammonium acetate. 300 cpm were spotted per lane

To verify that phosphorylation had occurred and to track the phosphoglucans at every experimental step with high sensitivity, radioactive labelling using [β-^33^P]ATP was performed. Then, an aliquot of the sample was measured by means of scintillation counting. However, as this only provides information about the radioactivity of the sample, not about the components, another aliquot was taken and used for TLC, and the distribution of the radioactivity was analysed by means of phosphor imaging. A mixture of ^33^P-labeled ATP, ^33^P-ADP, and ^33^P_i_ was used as a reference. Polyethylenimine cellulose TLC plates were used as negatively charged partner to interact with the immobilized positively charged ion groups, and the analytes were primarily separated according to their mass-to-charge ratio. This resulted in a very low mobility of ^33^P-ATP, whereas ^33^P-ADP and ^33^P_i_ migrate faster. However, phosphorylated α-glucan chains (and uncharged glucans) have a high mass-to-charge ratio, which causes them to hardly interact with the positive groups of the PEI TLC plate and to therefore migrate close to the running front, as shown in Fig. 3. It was therefore necessary to always re-buffer the phosphoglucans before TLC in order to prevent the acetate ions from interacting with the thin-layer plate and causing smearing. After isoamylase digestion, in addition to the phosphorylated chains, there was a large number of neutral chains and some ^33^P components as well, which would impair the analyses (Fig. 3). For the optimum TLC separation result, it was essential to prepare a fresh running buffer each time, as ammonium bicarbonate tends to decompose into ammonia, carbon dioxide, and water in the presence of moisture. Furthermore, to facilitate and improve the subsequent purification and analysis of the phosphoglucans, it is recommended to wash the starch very thoroughly to remove all unbound ³³P as well as possible. Moreover, depending on the starch type (different species or mutants), the number of washing steps needed to remove all the unbound ³³P may vary. Several approaches were tested to effectively separate the phosphoglucans from the neutral glucans and other phosphor components, with anion exchange chromatography proving to be the most accurate method. This was achieved by using Q Sepharose FF as the column matrix and eluting it with different concentrations of ammonium acetate. An aliquot of each collected fraction was used for the scintillation measurement. Here, it was important to pack the column correctly such that no air bubbles remained. Otherwise, the capacity of the Sepharose would be reduced and phosphorylated glucans could be lost. Subsequently, all fractions showing a radioactive signal were analysed in more detail by means of TLC, revealing the phosphoglucans at the running front. In order to confirm that the anion exchange chromatography did not result in any loss of phosphoglucans and still led to the separation of phosphorylated and neutral chains, the flow-through and the wash fraction were tested for radioactivity and glucans using liquid scintillation counting and iodine staining, respectively. Due to the low phosphoglucan concentration, the fractions were pooled according to the TLC results shown in Fig. 3 (lane 3–6; fractions of 0.02 M and 2–3 of 0.2 M ammonium acetate) and prepared for subsequent MS analyses. Division with regard to different amounts of ammonium acetate can also reflect specific subgroups of phosphorylated glucans and, therefore, can form a basis for their separation in further analyses.

### Analysis of phosphoglucans using MALDI–TOF mass spectrometry

Next, a part of the sample was treated additionally using BAM. Here, it is important to note that BAM’s action could be inhibited by increasing amounts of the catalytic end product maltose; thus, digestion must be carried out with sufficient volumes of incubation buffer and should be repeated twice. This step was validated by analysing the phosphoglucan pattern after several BAM incubations by means of MALDI-TOF MS, which revealed no detectable difference in the spectra after the second and third hydrolysis steps. The exoamylase releases maltose units from the non-reducing end until it reaches the vicinity of a phosphate residue, which it cannot pass. A shift from long to shorter chains is thereby obtained, which provides an approximation of the position of the phosphorylation with respect to the reducing and non-reducing ends when compared to the sample treated with isoamylase exclusively. To obtain more information regarding the phosphate position within the chains, the reducing ends of phosphoglucans were additionally labelled using 3AQ. Therefore, the AQ label represents the side of branching points.

**Fig. 4:**
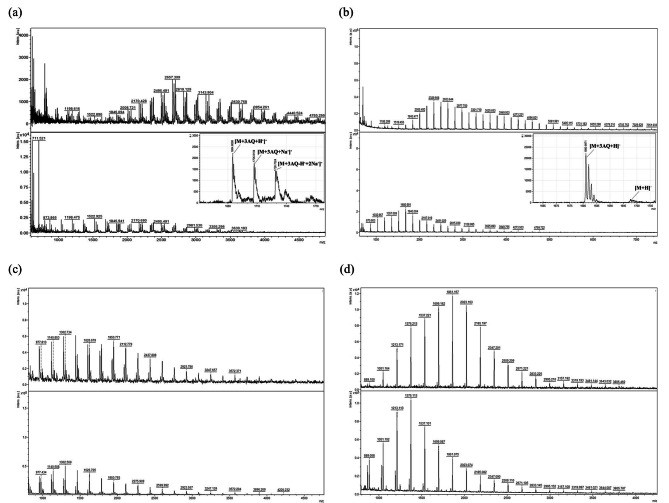
MALDI-TOF MS analysis of single phosphorylated glucans using maize* ae* starch. Starch was treated with GWD and ISA (**a**, **b** top) or incubated with GWD and ISA followed by BAM treatment (a, b bottom). Maize* ae* starch samples treated exclusively with ISA (**c**, **d** top) and maltodextrin (**c**, **d** bottom) were utilized as negative controls, and the spectra were recorded in positive (**c**) and negative (**d**) modes. The reducing ends of the phosphoglucans were labelled with 3AQ and analysed by means of MALDI-TOF MS. (**a**) The spectra were recorded in the positive reflector mode. The following equation was used for calculations: m/z= (DP*162) + 3AQ + HPO₃⁻ + H⁺. (**b**) The spectra were recorded in the negative reflector mode. The equation m/z= (DP*162) + 3AQ + HPO₃⁻ - H⁺ was used

The samples were analysed using MALDI-TOF MS. The spectra recorded using the positive and negative reflector modes display the distribution of single phosphorylated glucans after ISA treatment (Fig. 4a and b, top) and the distribution of single phosphorylated chains after ISA and BAM digestion (Fig. 4a and b, bottom). On the one hand, these spectra demonstrate that GWD does not utilize special DPs for phosphorylation; otherwise, no distribution would be visible, only individual peaks. Therefore, a similar width distribution as for neutral glucans was observed. On the other hand, a clear shift to shorter chains was generated after BAM incubation, which clearly proves that the non-reducing ends were not occupied by phosphate groups, so that the exoamylase was able to act. However, when comparing the spectra recorded in positive and negative ion modes, it became obvious that they differed in quality and quantity, especially those after ISA hydrolysis only. Single phosphorylated chains were detected in the ranges of DP6-44 (after ISA) and DP3-28 (after ISA and BAM) in negative ion mode and in the ranges of DP6-28 (after ISA) and DP3-21 (after ISA and BAM) in positive mode. This indicates that the long phosphoglucan chains are more difficult to cationise than short ones, as also shown by the signal-to-noise ratio and intensity obtained. The complexity of the spectra differed massively, with H⁺ and Na⁺ adducts being detected in the spectra measured in the positive mode, whereas in the negative ion mode, the phosphoglucans were exclusively single deprotonated. However, metastable ions also formed, identifiable by the absence of an isotopic pattern (Fig. 4b). These fragmentation events were likely favoured by the reducing end labels. Moreover, in positive mode, the described adducts were found with an additional exchange of a proton with a sodium ion, which most likely took place at one of the hydroxyl groups of the phosphate residues, considering chemical properties such as acidity and pH value (around 5.5) of the matrix used. Phosphate has three pka values (2.2, 7.2 (5.8 as an ester), and 12.4 [27], whereas glucose theoretically has six pka values, all above 12. Overall, the spectra show the success of the anion exchange chromatography, as no neutral chains were detected.

**Fig. 5:**
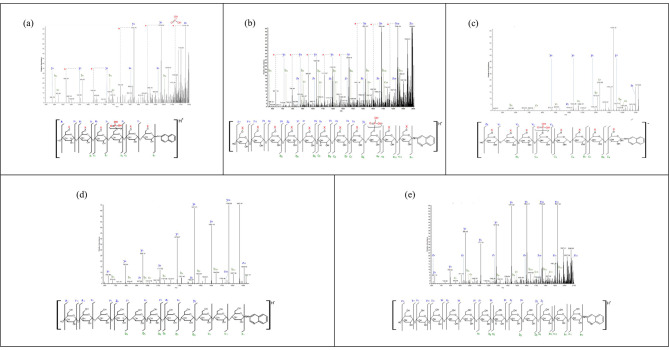
MALDI-MS/MS analysis of single phosphorylated glucans using maize *ae* starch. Following in vitro phosphorylation, starch was treated with ISA to release glucan chains, and the reducing ends of glucans were labelled with 3AQ. (**a**) In addition to the isoamylase digestion, a BAM treatment was carried out to remove maltose units from the non-reducing end. Single phosphorylated glucans with a mass of 1197 m/z (DP6) were fragmented by means of CID in positive mode. The spectrum displays glucan chains from DP1 to DP5, which carry a single phosphate group (y-fragments), and chains from DP4 to DP6, with one phosphoesterification but a loss of water (z-fragments). Neutral chains were found where phosphate elimination had occurred, indicated by red asterisks. b-and c-fragments, which do not have a reducing end label, were also observed. (**b**) The spectrum shows the fragmentation of DP11 (2007 m/z) with a single phosphate group in the range of DP3-DP10 (y-fragments). The loss of the phosphate group is marked by a red asterisk. Fragmentation was run in positive mode. (**c**) Fragmentation of DP8 with a single phosphate group (1519 m/z) was performed using CID in negative mode; y-fragments in the range of DP4-DP7 were detected. (**d**) Starch from maize ae was only treated with isoamylase, without previous GWD phosphorylation. The fragmentation spectrum displays neutral chains in the ranges of DP3-DP10 (y-fragments) and DP4-DP11 (b-fragments). The spectrum was recorded in positive mode. (**e**) Maltodextrin was also fragmented using CID in positive mode

With MALDI-TOF MS, it is possible to make a statement about the distribution of phosphorylated glucans, but no information about the exact locations of the phosphate groups within the chains is obtained. Therefore, the next step was to implement MALDI-MS/MS. The phosphoglucans were labelled at the reducing ends with 3AQ and fragmented by means of collision-induced dissociation (CID) in positive ion mode. A few examples of typical fragmentation patterns are depicted in Fig. 5. As negative controls, maltodextrin (Fig. 5e) and maize *ae* starch were used. These were digested exclusively with ISA; GWD was omitted (Fig. 5d). The resulting fragment ion patterns of the phosphoglucans were compared with those of the negative controls, demonstrating for both the phosphorylated and neutral glucans that CID particularly induces cleavage of the glycosidic bonds (b- and c-fragments, x- and z-fragments). b- and c-fragments describe the orientation of the glucans from the non-reducing ends, while x- and z-fragments describe the same from the reducing ends. With the use of the AQ label, a precise assignment of the fragments was achieved. However, the x- and z-fragment ions of the phosphoglucans displayed a mass difference of 80 m/z when compared to those of the neutral ones (Fig. 5a, b, d, e), which can be assigned to one phosphate that split off from the glucans. The fragmentation of phosphoglucans with DP6 (parent ion 1197 m/z) following BAM hydrolysis is shown in Fig. 4a. The detected peaks correspond to fragments with 3AQ labels in the range of DP1-5 (y-fragments) and fragments of DP4-6 that lost water (z-fragments). b- and c- fragments were also found in the spectrum, but these provide little information to describe the exact position of the phosphate group within the glucan chain, as there they have no reference point or start. Furthermore, neutral chains were observed where phosphate elimination took place, as indicated by red asterisks. Similar findings were obtained for phosphoglucans with DP11 (after ISA only, parent ion 2008 m/z; 5b); y-fragments of DP3-10 and z-fragments of DP4-11 were observed, as well as the phosphate group cleavage. However, both MS/MS spectra show that a mixture of isomeric glucans was present, and there was no specific phosphorylation location within the glucans. It seems that the phosphate group restricts the action of BAM more distantly below a certain DP length, i.e., a phosphate group directly at the non-reducing end inhibits BAM, but so do phosphate residues near the reducing end of short chains. Because repeated incubation was performed to exclude partial BAM hydrolysis, there is no explanation as to why y1 to y5 were detected with a phosphate residue after the fragmentation of DP6. It has been demonstrated for neutral glucans that ΒΑΜ is able to digest glucans longer than DP3 [28, 29]; hence, phosphorylation within the glucan somehow shifts the minimum length of glucans that can be hydrolysed by the exoamylase. Since the phosphorylated chains can be ionized in negative mode more easily in MALDI-TOF MS, this was also tested for MS/MS. A typical fragmentation spectrum recorded in negative mode of DP8 with a single phosphate residue is depicted in Fig. 4c. Here, DP4-7 for y-fragments and z8 were observed, along with several b- and c-fragments, which have only little information content. Moreover, no peaks for phosphate elimination could be identified; thus, in general, it can be stated that fragmentation in the positive ion mode provides significantly more information regarding the phosphate group position within the chains.

### Advantages and limitations of MALDI-TOF MS

MALDI-TOF MS analysis was used in this study to obtain a quick and easy overview of the distribution of single phosphorylated chains, showing that the negative ion mode is better suited to ionizing long chains as well. The rapid acquisition of different distribution patterns of carbohydrates is one of the major advantages of this method, along with its high mass accuracy and the low cost of supplies after the initial device purchase. Furthermore, MALDI is a very gentle ionization technique; in general, the formed molecule ions still have a low internal energy, which enables the monitoring of intact molecules. Nevertheless, in-source and/or post-source fragments also appear at certain laser intensities. These could be used for further structural analyses of particular glycans, as information complementary to the CID results could be obtained. Another advantage of this ionization method is that mainly singly charged ions are generated and not multiply charged ones, as in the case of, for example, ESI. However, MALDI-TOF MS cannot be performed directly on phosphorylated starch granules. Therefore, several additional processing steps are required due to the low amount of phosphoglucans. Nevertheless, after the hydrolase treatment and enrichment steps applied in this study, the identification of single phosphorylated α-glucan chains became feasible. A limitation that should be mentioned is that MALDI is only semi-quantitative. However, techniques such as HPLC, HPAEC-PAD or capillary electrophoresis could be used here. These would be suitable methods for precise quantification. The phosphorylated chains could be analysed after hydrolysis with ISA and/or BAM and an approximate estimation of the phosphate group within the glucan chain would be given. Nevertheless, a clear assignment is not feasible. Mass spectrometry is the method of choice here, as it provides clear evidence that the glucans are phosphorylated *via* the mass change and not only variations in the elution times. Furthermore, using MS/MS analysis, additional structural details can be obtained. Applying CID led to the cleavage of the glycosidic bonds, in particular where different DPs are formed, each of which carries a phosphate residue. Other fragmentation methods were also tested, such as PQD and LID, but the recorded spectra contained significantly less information when compared to CID. Some y- and z-fragments were also generated, but with considerably reduced efficiency. Furthermore, the loss of phosphate groups could not be detected. By additionally labelling the reducing ends, it was possible to determine the position of the phosphate group within the glucans. However, the selected parent ions always represented a mixture of isomeric phosphorylated chains; in addition, the phosphate groups appear to have a large-scale influence on BAM’s action below a certain chain length. Thus, ion mobility spectrometry–mass spectrometry (IMS-MS) is the technique of choice for future experiments.

## Conclusion

The maximum possible degree of phosphorylation can vary greatly depending on the starch origin. However, the method demonstrated here enables the analysis of all starches, whereby certain only minor adjustments such as the incubation time and number of washing steps have to be made. In general, the protocol established herein paves the way to understanding further processes of starch phosphorylation and, thus, to describing the parameters of starch for various applications and elucidating the entire process of starch metabolism in greater detail. In further studies, the phosphorylation patterns of other starch-relevant dikinases—such as PWD, which phosphorylates the C3 position of amylopectins—could be examined, in addition to how other starch-metabolism-related proteins and enzymes influence these patterns. The analysis of phosphoglucans could be extended by using BAMs and ISAs from various species; this could reveal an impact on the phosphorylation pattern and could help us to understand the phosphorylation mechanism in greater depth.

The whole procedure described in this study provides information about starch interacting protein/enzyme properties, as well as overall starch characteristics. More generally, this procedure also allows the detailed analysis of other phosphorylated glucans/glycans and oligosaccharides, even when they are only present in small quantities.

## Data Availability

Data is provided within the manuscript.

## Abbreviations

3AQ: 3-aminoquinoline
ACN: Acetonitrile
BAM: β-amylase
BSA: Bovine serum albumin
CA: p-coumaric acid
CID: Collision Induced Dissociation
Cv: Column volume
DP: Degree of polymerization
DTE: Dithioerythritol
ae: Amylose extender
GWD: α-glucan, water dikinase
IMS-MS: Ion Mobility Spectrometry–Mass Spectrometry
ISA: Isoamylase
LID: Laser Induced Dissociation
MALDI-TOF: Matrix Assisted Laser Desorption/Ionisation-Time Of Flight
MS/MS: Tandem mass spectrometry
PQD: Pulsed Q Collision Induced Dissociation
PWD: Phosphoglucan, water dikinase
